# Supplementary figures and images for: Reln haploinsufficiency enhances fentanyl-induced locomotion and striatal activity without affecting opioid reinforcement and relapse-like behavior
Source: bioRxiv. 2026 Mar 18:2026.02.21.707172. Preprint. [Version 3] doi: 10.64898/2026.02.21.707172 (PMC13015708; doi:10.64898/2026.02.21.707172)

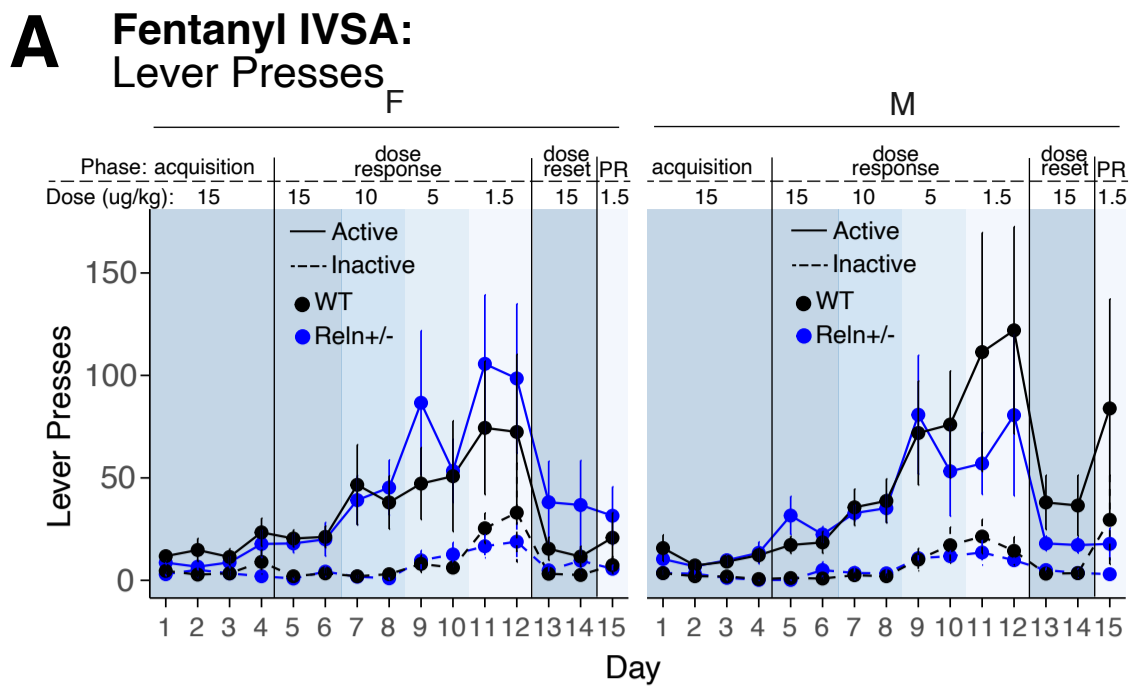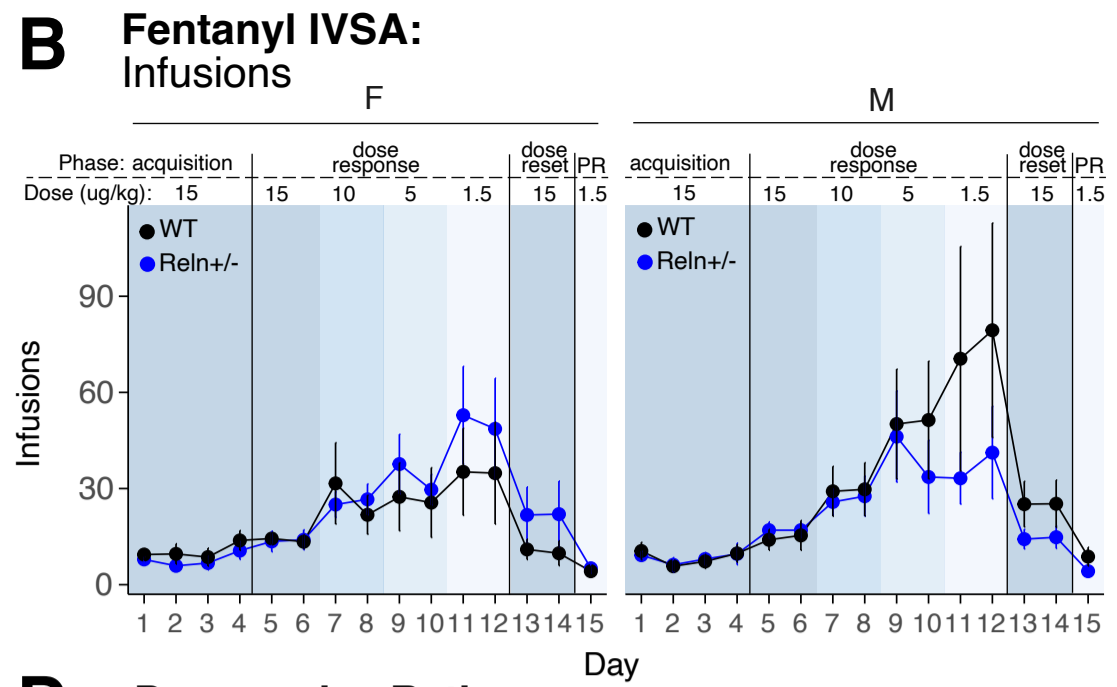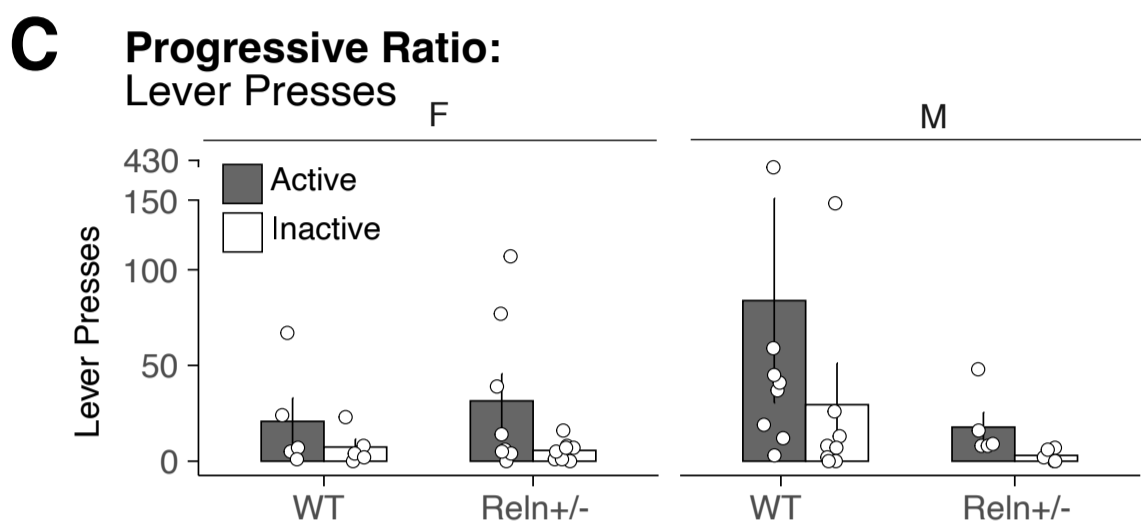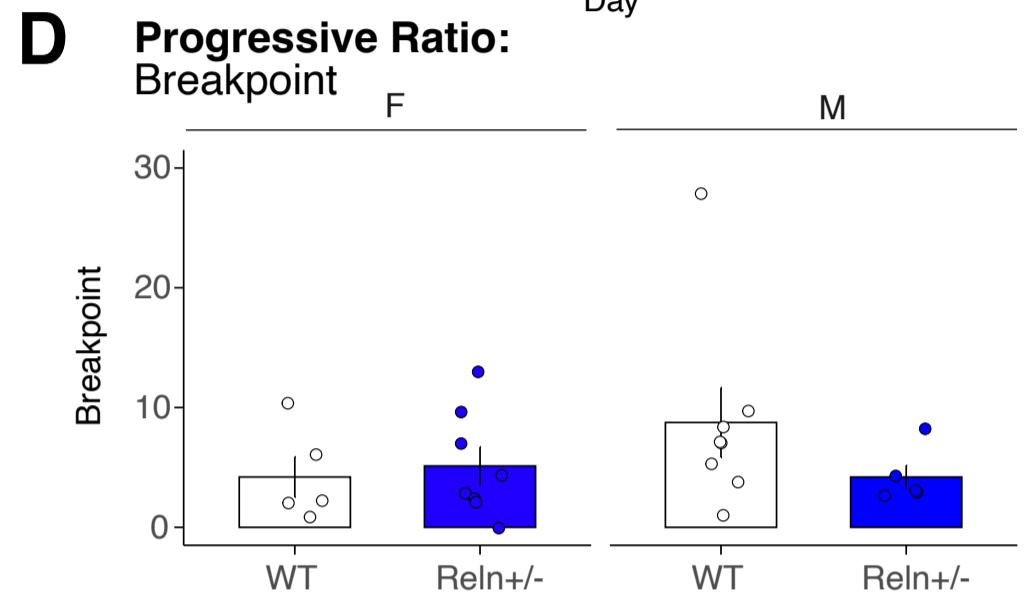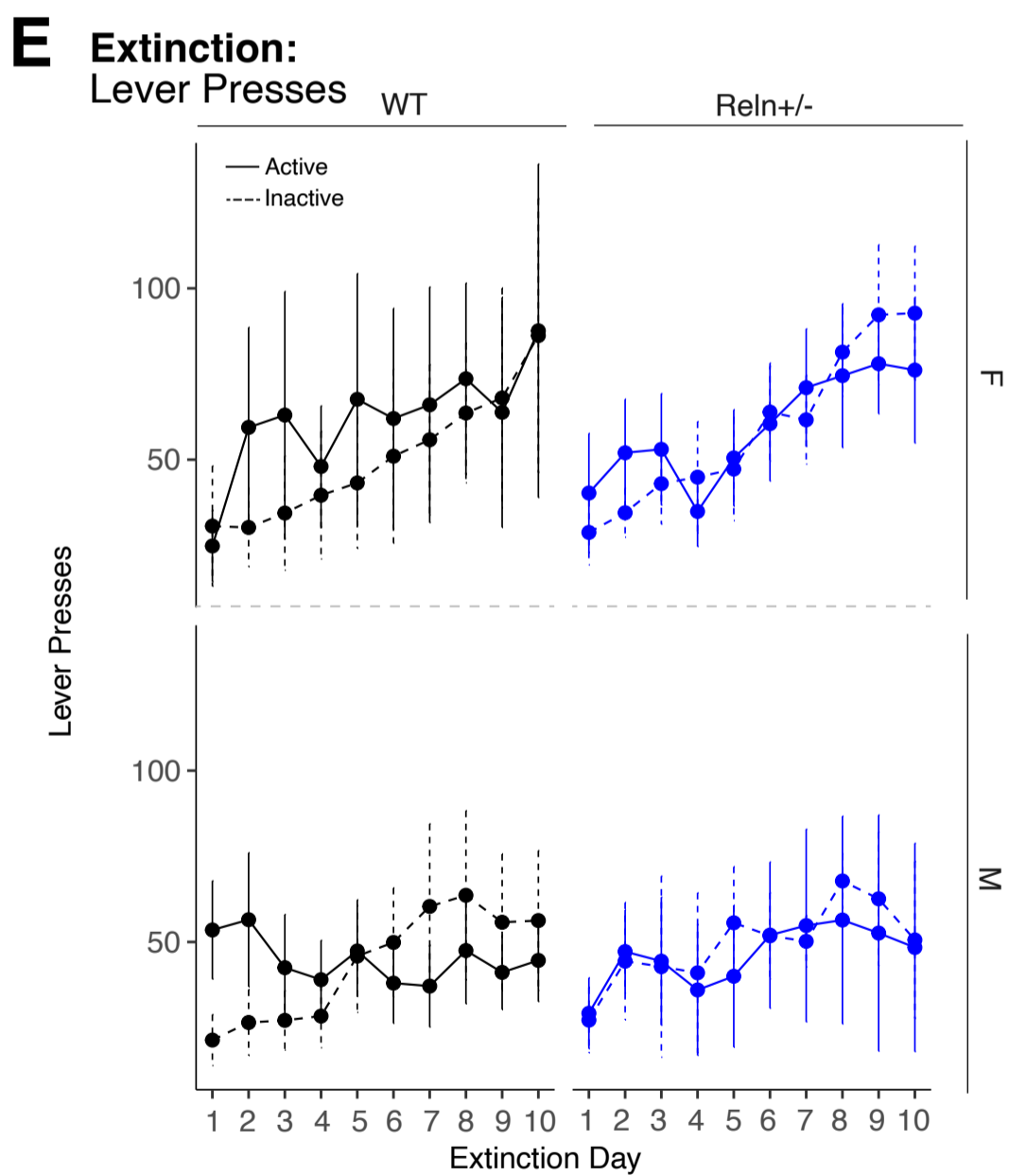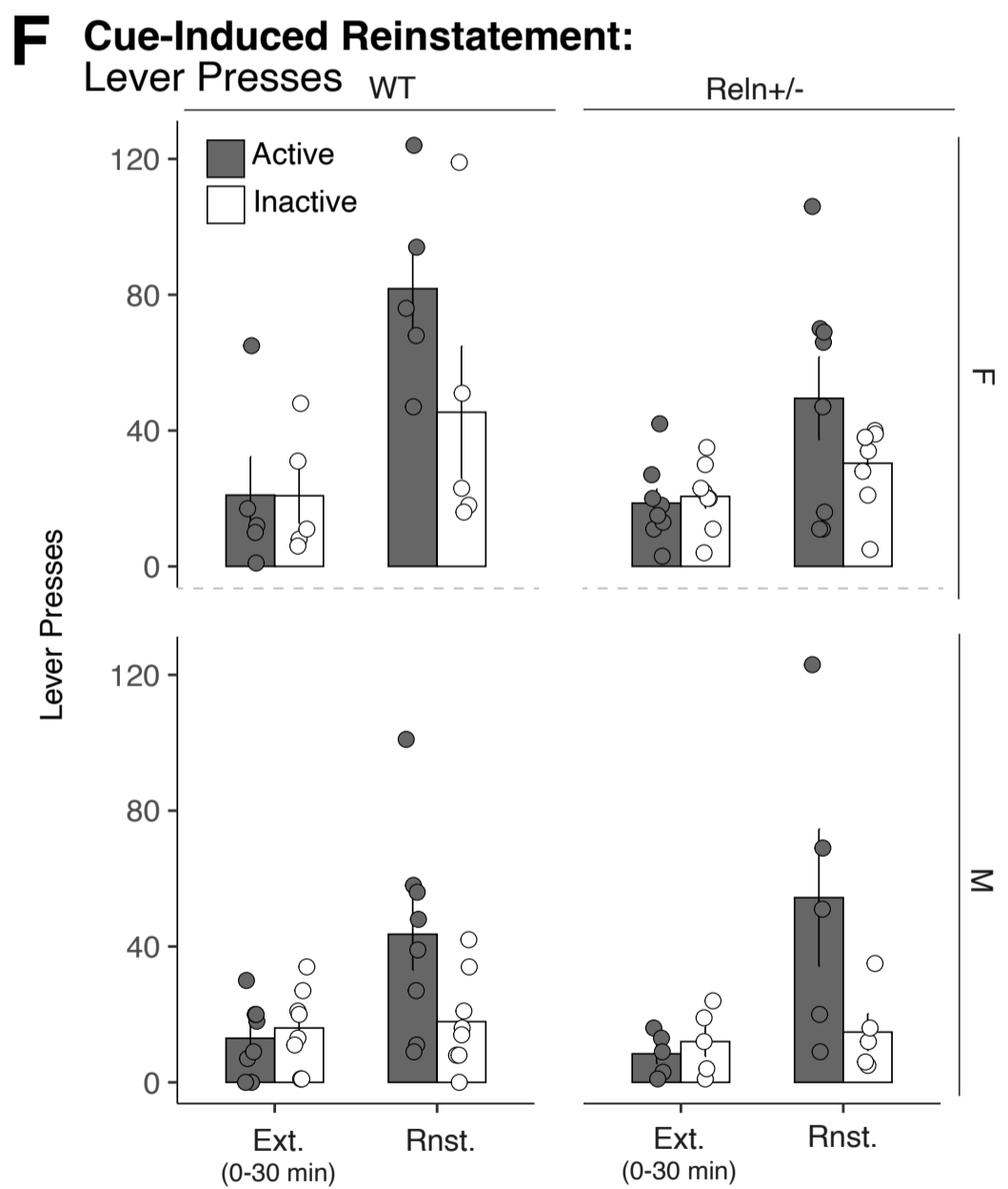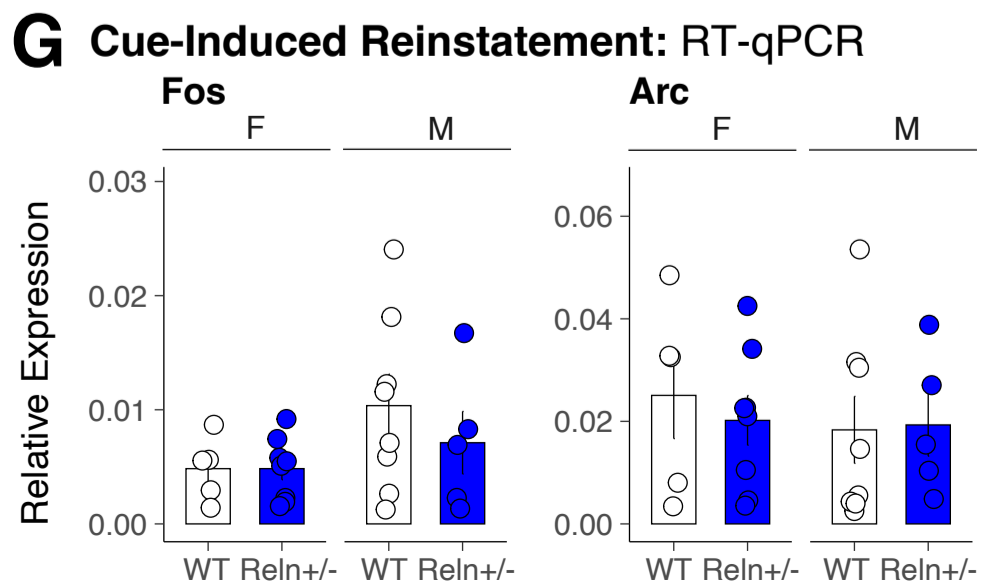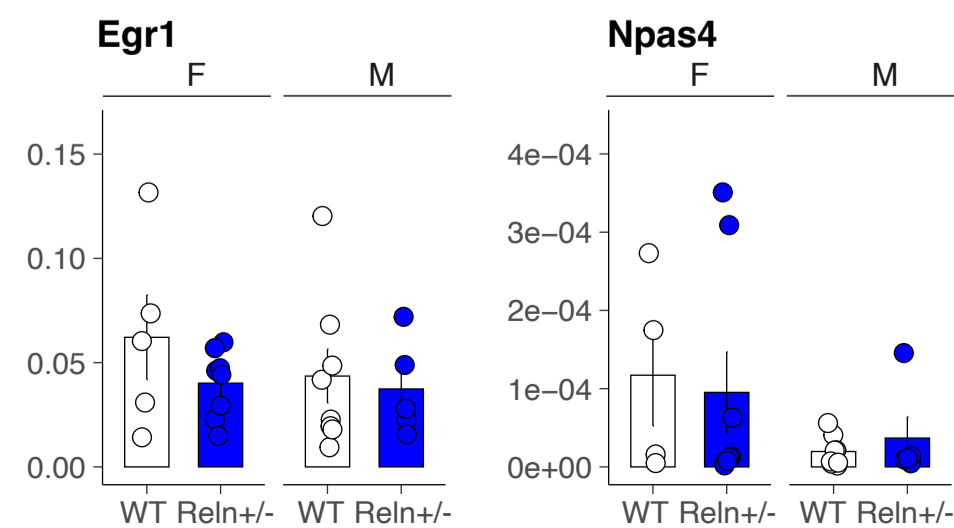

Supplement: Supplement 2 — (A-B) Active (solid line) and inactive (dashed line) lever presses (A) or infusions (B) across all IVSA sessions (Day 1–15), stratified by sex and genotype. (C-D) Total lever presses (C) or breakpoint (D) during the progressive ratio session, faceted by sex and genotype. (E) Active (solid line) and inactive (dashed line) lever presses during extinction phase (Extiction, Day 1–10), faceted by sex and genotype. (F) Comparison of active and inactive lever presses during the first 30 minutes of the last extinction session (Ext., Day 10) and during the subsequent 30-minute cue-induced reinstatement session (Rnst), faceted by sex and genotype. (G) Relative expression (2^-ΔCt) of Fos, Arc, Egr1, and Npas4 expression in the dorsal striatum following cue-induced reinstatement, normalized to Gapdh; faceted by sex and genotype. Data are mean ± SEM. Female: WT n=5, Reln+/− n=8; Male: WT n=8, Reln+/− n=5. [file media-2.pdf]

# A Fentanyl CPP

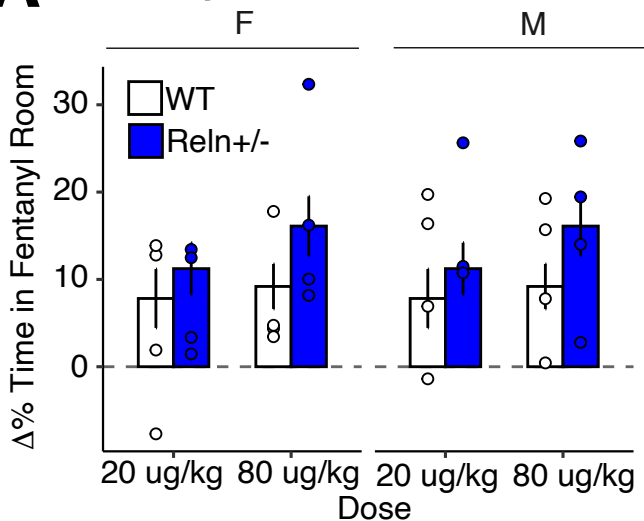

# B Fentanyl-induced Locomotion

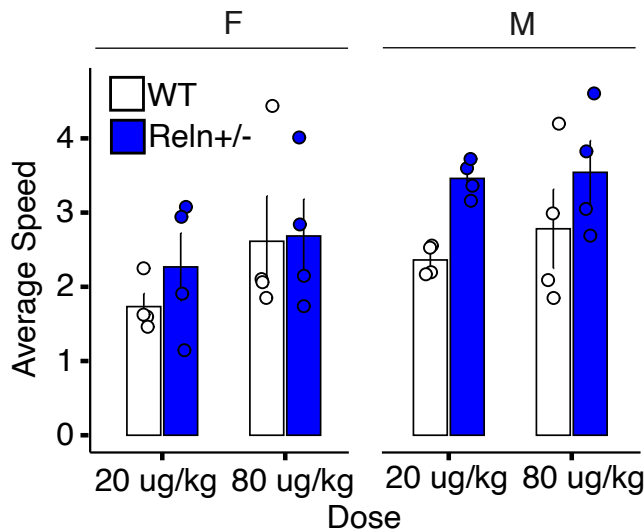

Supplement: Supplement 4 — (A) Change in percent time spent in the fentanyl-paired room on the test day relative to baseline, for mice conditioned at 20 μg/kg or 80 μg/kg, faceted by sex and genotype. (B) Average locomotor speed (total distance / total time) during the first CPP conditioning session following acute fentanyl injection at 20 or 80 μg/kg, faceted by sex and genotype. Data are mean ± SEM. Female: WT n=4; Reln+/− n=4; Male: WT n=4, Reln+/− n=4. [file media-4.pdf]
