# Supplementary material for: Reln haploinsufficiency enhances fentanyl-induced locomotion and striatal activity without affecting opioid reinforcement and relapse-like behavior
Source: bioRxiv. 2026 Mar 18:2026.02.21.707172. Preprint. [Version 3] doi: 10.64898/2026.02.21.707172 (PMC13015708; doi:10.64898/2026.02.21.707172)
Supplement: Supplement 3 — (A-C) Total fentanyl intake (ug) averaged across the two sessions at each unit dose during the dose response phase for WT (n=13, black) and Reln+/− (n=13, blue) mice (A) or separated by females (B; WT n=5, Reln+/− n=8) and males (C; WT n=8, Reln+/− n=5). Data are mean ± SEM. [file media-3.pdf]

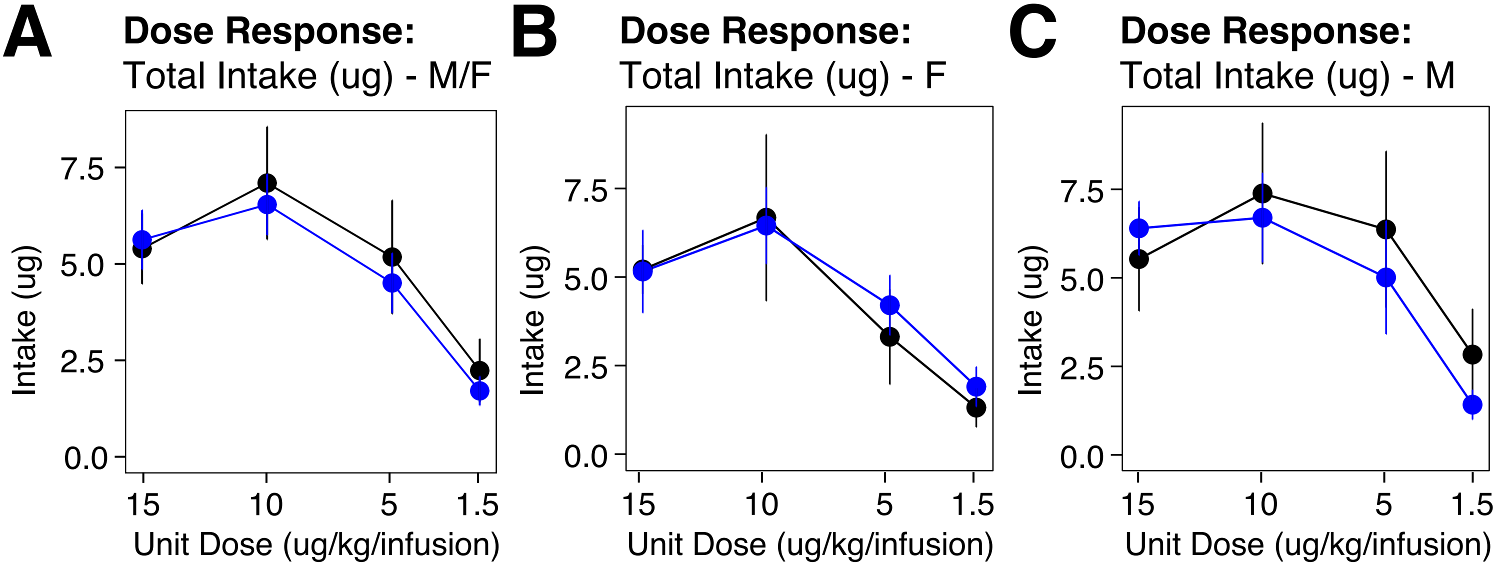

A
Dose Response:
Total Intake (ug) - M/F
Intake (ug)
0.0
2.5
5.0
7.5
15
10
5
1.5
Unit Dose (ug/kg/infusion)
B
Dose Response:
Total Intake (ug) - F
Intake (ug)
0.0
2.5
5.0
7.5
15
10
5
1.5
Unit Dose (ug/kg/infusion)
C
Dose Response:
Total Intake (ug) - M
Intake (ug)
0.0
2.5
5.0
7.5
15
10
5
1.5
Unit Dose (ug/kg/infusion)
